# A Simulation Optimization Approach to Epidemic Forecasting

**DOI:** 10.1371/journal.pone.0067164

**Published:** 2013-06-27

**Authors:** Elaine O. Nsoesie, Richard J. Beckman, Sara Shashaani, Kalyani S. Nagaraj, Madhav V. Marathe

**Affiliations:** 1 Network Dynamics and Simulation Science Laboratory, Virginia Bioinformatics Institute, Virginia Tech, Blacksburg, Virginia, United States of America; 2 Department of Computer Science, Virginia Tech, Blacksburg, Virginia, United States of America; College of Medicine, Hallym University, Republic of Korea

## Abstract

Reliable forecasts of influenza can aid in the control of both seasonal and pandemic outbreaks. We introduce a **sim**ulation **op**timization (SIMOP) approach for forecasting the influenza epidemic curve. This study represents the final step of a project aimed at using a combination of simulation, classification, statistical and optimization techniques to forecast the epidemic curve and infer underlying model parameters during an influenza outbreak. The SIMOP procedure combines an individual-based model and the Nelder-Mead simplex optimization method. The method is used to forecast epidemics simulated over synthetic social networks representing Montgomery County in Virginia, Miami, Seattle and surrounding metropolitan regions. The results are presented for the first four weeks. Depending on the synthetic network, the peak time could be predicted within a 95% CI as early as seven weeks before the actual peak. The peak infected and total infected were also accurately forecasted for Montgomery County in Virginia within the forecasting period. Forecasting of the epidemic curve for both seasonal and pandemic influenza outbreaks is a complex problem, however this is a preliminary step and the results suggest that more can be achieved in this area.

## Introduction

Influenza continues to be one of the most important human infectious diseases; responsible for thousands of deaths in the United States each year. In April of 2009, a novel influenza A virus emerged in Mexico and the United States. Although the 2009 H1N1 influenza pandemic was milder than expected, the emergence of the novel virus reinforced the need to improve tools for analyzing surveillance data and forecasting for decision making during a pandemic [1]. Mathematical and computational models are used as tools to aid pandemic planning. Specifically, individual-based epidemiology models are useful in evaluating the possible effectiveness and economic impact of different response strategies [2]–[6].

This study extends the application of the individual-based epidemiology model to forecasting of the epidemic infection curve (hereafter referred to as the epidemic curve). The epidemic curve is defined as the daily or weekly number of cases observed for the duration of the epidemic [7]. We seek to forecast the time at which the epidemic peaks, the number of infected individuals at the peak and the cumulative infected counts. These measures provide a summary of the epidemic curve and are important to public health officials. An accurate forecast of these measures at a regional level would enable local public health officials to evaluate intervention strategies and make educated decisions during an influenza epidemic [8]–[10].

Real-time forecast of the epidemic curve requires a combination of good monitoring systems and adequate assumptions about the disease model parameters [9], [11]. Conventional methods for monitoring influenza-like illness (ILI) and acute respiratory tract infections from general practices, family doctor and government clinics are being used in many countries 11–18. These methods were also used to monitor influenza activity during the 2009 H1N1 pandemic [19]–[21]. In addition, several methods have been proposed for real-time modeling and forecasting of epidemic dynamics [9], [11], [22]–[24]. Hall et al. [24] proposed using a deterministic compartmental model to estimate epidemic dynamics. Their method was used to retrospectively predict the amplitude and durations of three pre-2006 influenza pandemic events in England and Wales. They used regression techniques to fit a time-series disease incidence curve obtained from a traditional differential equation epidemiology model to the mortality and influenza-like illness (ILI) data for the three pandemics. This technique required estimation of nine parameters, including the reproduction number. The model also assumed knowledge of the natural history of the disease from detailed epidemiological studies in the early stages of the pandemic.

Hsieh and Cheng [25] demonstrated the use of a variation of a single-equation Richards model to estimate outbreak severity. Their method used a power-law logistic equation to estimate parameters based on the epidemic curve. The method was applied to the multiphase 2003 severe acute respiratory syndrome (SARS) outbreak in Toronto. Hsieh [26] also employed the same model to estimate parameters for the 2009 H1N1 influenza pandemic in six countries in the southern hemisphere. Similarly, Nishiura presented a discrete time stochastic model for forecasting the 2009 H1N1 pandemic [23]. To retrospectively forecast the pandemic in Japan, a likelihood-based approach was used in parameter estimation. Ohkusa et al. [23] also used a simple SIR model for forecast during the pandemic. In contrast, Ong et al. [11] described a real-time system to both monitor and forecast different epidemic outcome measures in Singapore during the 2009 pandemic. The surveillance system collected data on ILI instances from twenty three participating general practice and family doctor clinics in Singapore. Since H1N1 had low hospitalization and mortality rates, the study did not use hospital and fatality data. A stochastic compartmental model with particle filtering was used in real-time epidemic incidence forecast. ILI data collected at general practice and family doctor clinics in Singapore was refitted each day to provide sequential updates on forecasts.

All previously discussed approaches to forecasting use either a variation of the differential equation epidemic model or a region-dependent disease transmission model, or both, making it difficult to model for changes in human mobility and interaction patterns. In contrast, Chao et al. [9] used a stochastic epidemic simulation model, which includes descriptions of interactions between individuals (with demographic information) at different mixing groups (schools, homes, work etc.). Forecasts of the characteristics of the 2009 influenza pandemic in addition to the potential effects of interventions were made for Los Angeles (LA) county. The forecasting process combined the stochastic model with a monitoring system established by the LA county department of public health. The stochastic epidemic simulation model used by Chao et al [9] is similar to that used in this study. Both models seek to represent individuals and interactions between individuals. However, there are differences in the data sources, the method of constructing the networks and some of the assumptions in the disease model. There are also differences in the manner in which the models are used in forecasting. In this study we present an approach which combines an individual-based model and an optimization technique to recursively estimate model parameters and forecast the epidemic curve as data is sequentially updated during an epidemic. Shaman et al [27] employed a similar approach based on an assimilation of various models to achieve forecasts of the peak time seven weeks in advance. In this study, we seek to forecast the peak time in addition to the expected peak infected and total infected population. To our knowledge, the approach presented in this paper has not been previously studied.

### Approach

Given an epidemic, let 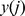 represent the number of new cases on day 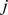. The time series 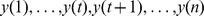 denotes the number of new cases observed each day for the duration of the epidemic, 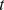 indicates the day of forecast and 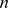 is the expected duration of the epidemic. Note that precise values of the 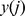
's and 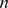 are unknown.

The problem can be formerly defined as follows: given the state of the epidemic on day 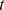 as described by 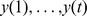, we seek to predict some function 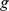 of 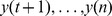. We focus on three measures:

Peak Time: 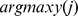 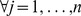,

Peak Infected Count: 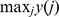 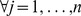 and

Total Infected Count: 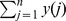.

These selected measures are useful for estimating epidemic impact and decision making regarding selection and introduction of control measures for optimal effectiveness [8], [10].

### Overall Process

**Figure 1:**
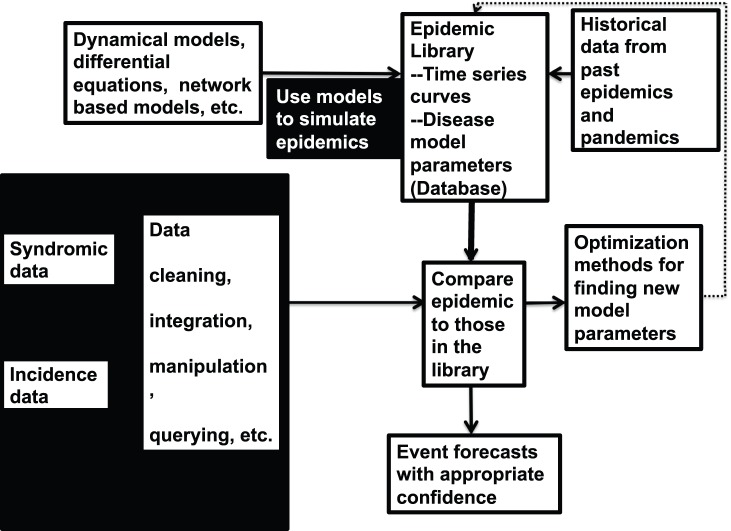
Summary of methodology. We develop a library of past and simulated epidemics. Given surveillance data for a current epidemic, we compare the partial epidemic curve to those in the library. The novel epidemic is either assigned to a case in the library or identified as being different from those in the library. If the epidemic is different from those in the library, we estimate the model parameters, forecast the epidemic curve and update the library.

This study represents the final step of a project aimed at using a combination of simulation, classification, statistical and optimization techniques to forecast the epidemic curve and infer underlying disease model parameters (Figure 1). During an epidemic, ILI or other forms of surveillance data can be obtained from sources such as the United States Centers for Disease Control and Prevention (CDC), FluNet, Distribute Project, etc. Given the availability of surveillance data, we describe the process as follows. First, we build a library of past and simulated epidemics. Simulated epidemics are replicated several times to capture the variability in the system. Using a classification approach, we propose a parameter set to model a new outbreak at time 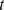 based on available data up to time *t*. We use random forest; a supervised tree-based classification method to assign the new epidemic to an existing case in the library. Random forest is efficient on large databases, tends to achieve a high accuracy on most classification problems and enables estimation of importance variables, which is especially useful for data sets with many variables [28]. The efficacy of random forest in classification of partial epidemic curves was illustrated in [29]. If the match suggested by random forest is considered suitable, then the parameters of the epidemic in the library are used in modeling the new outbreak. On the contrary, if none of the epidemics in the library is deemed a good match, then we recursively apply a combination of simulation and optimization methods to propose new parameters.

### Study Objective

In this study, we focus on the event that the epidemic cannot be classified to any of the cases in the library (Figure 1). We therefore seek to estimate model parameters to forecast at time 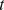 based on the epidemic curve up to time 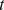. The **si**mulation **op**timization (SIMOP) algorithm introduced in this study employs the Nelder-Mead simplex method for optimization and an individual-based model for simulations. These methods are discussed in later sections and in the Supporting Information S1 file.

The forecasting procedure is repeated each day for the duration of the epidemic. Nonetheless, forecasts made before the peak of the epidemic are most preferred. Upon identification of a parameter set for modeling the outbreak, the individual-based model is used to investigate the effectiveness of various intervention measures and the effects of changes in individual behavior during the epidemic [30], [31]. However control measures are not presented in this study. This preliminary study is to validate and verify the forecasting method. In this study, we present forecasts for a baseline scenario and focus on epidemics with a single peak. Nevertheless, the methods can be applied to study situations in which a second peak (wave) is observed during an epidemic.

The proposed method is tested on simulated data. Simulated influenza incidence data is used as follows: the epidemics are simulated over synthetic social networks representing Montgomery County (MC) in Virginia, Miami and surrounding metropolitan regions (Miami), and Seattle and surrounding metropolitan regions (Seattle). Studying simulated epidemics for regions with demographic and rural-urban differences enables a thorough illustration of the methods' performance. The aims of this study are therefore to: (i) forecast the epidemic curve by forecasting the time to peak, peak infected counts and total infected counts, (ii) compare forecasts for epidemics simulated across different social networks, and (iii) forecast epidemics with different *noise* levels.

## Methods and Models

The process of parameter estimation using an optimization approach is similar to other proposed approaches based on least-squares. However, the novelty of this work lies in (i) the creation of the disease library, (ii) the flexibility in the approach such that it can be applied to forecasting using both a complex individual-based model and a simple SEIR differential equations model, and (iii) the general applicability of the approach to any time series data. The methods and SIMOP procedure are described.

### Disease Models and Parameters

The aim is to capture the shape of the epidemic curve by forecasting certain characteristics of the curve. We therefore estimate model specific parameters to accomplish this aim.

#### Individual-based model

The three model parameters estimated in this study are the disease transmissibility, incubation and infectious period distributions (see Table 1 for definitions). The transmissibility of a disease is typically represented using measures such as the reproduction number or the household secondary attack rate [32], [33]. The attack rate is the cumulative infection incidence observed within a population over the span of an epidemic. If the time of infection is known, the incubation duration can be derived. The infectiousness typically differs for different individuals due to factors such as age, symptoms and health state [6]. The incubation and infectious period parameters are therefore represented using discrete probability distributions.

**Table 1:** Parameter Definitions.

Parameters	Definitions	Example
Transmissibility	The rate at which disease propagates through the population	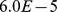 per sec/unit of contact time
Incubation Period	Duration between infection and onset of symptoms	0∶0.0 1:0.3 2:0.5 3:0.2
Infectious Period	Period during which infected persons can shed the virus	2∶0.0 3:0.3 4:0.4 5:0.2 6:0.1

The incubation (infectious) period is defined as follows: 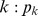 where 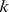 is the duration and 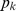 is the probability that an infected (infectious) individual will have an incubation (infectious) period of 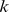 days. The disease transmissibility is given as the probability of infection per unit of contact time between a susceptible and infectious individual in the network.

The individual-based model consists of a dynamic social contact network and a disease model as discussed in a later section and in the SI file. The parameters estimated in this study are part of the disease model. In order to estimate these parameters, we make the following assumptions: (i) the **S**usceptible, **E**xposed, **I**nfectious and **R**ecovered (SEIR) model is sufficient to describe disease transmission and progression. (ii) The possible durations of the incubation and infectious periods are fixed as shown in Table 1. We therefore focus on estimating the probabilities of observing each incubation (infectious) duration in the network. (iii) The network is assumed to remain unchanged during the course of the epidemic implying new individuals do not enter or leave the synthetic population. (iv) Biological differences between age groups are not represented. (v) When dealing with a novel epidemic, the prior immunity in the population is assumed to be minimal or null. These assumptions appear sufficient for illustrating the method.

### Forecasting Algorithm

The SIMOP procedure can be described in three steps:

*Step i*: initialize the individual-based model and the Nelder-Mead simplex method,

*Step ii*: run the Nelder-Mead algorithm to find new parameter sets,

*Step iii*: simulate epidemic using the proposed parameter set and evaluate the objective function.

*Steps ii* and *iii* are repeated until convergence. We describe methods and processes involved in fulfilling each of these steps.

#### Step i: Initializing the SIMOP algorithm

We select initial parameters for both the epidemic model and the Nelder-Mead algorithm. The initial parameters used in the Nelder-Mead algorithm are crucial to the optimization process. For the first day (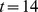) of forecast, we randomly sample eleven parameter sets from the disease library because Nelder-Mead algorithm requires 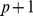 initial parameter sets where 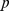 is the number of parameter values. The eleven parameter sets at convergence at time 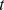 are used to initialize the procedure for forecasts at time 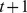. The procedure is carried out in this manner since the number of infected at time 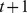 is dependent on the number infected at previous time steps 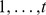. The parameter sets in the library are similar to those used in modeling seasonal influenza epidemics and the 2009 H1N1 pandemic [5], [34]. We also use parameters from a sensitivity analysis study presented in [35].

For the purpose of this study, the initialization process for the individual-based model involves selecting a social network, choosing the number of persons to initially infect, setting an upper bound on the epidemic duration, and defining a disease model.

#### Steps ii: Estimating parameters

As stated, the individual-based model and the Nelder-Mead simplex method are used in the SIMOP algorithm. The Nelder-Mead simplex algorithm is used to propose new parameters. The parameters are then used in simulating epidemics using the individual-based model. This process is repeated several times until the algorithm converges as discussed in the proceeding section.

The Nelder-Mead method was selected after comparing its performance (accuracy, computational time and cost) to Simulated Annealing [36] and the classical stochastic root finding approach in Robbins and Monro [37]. The method serves as an illustration that similar optimization techniques can be used in combination with simulations to solve the problem of forecasting the epidemic curve. The Nelder-Mead algorithm is also easy to implement and modify. We do not claim that the Nelder-Mead is the best possible optimization method that can be used in such a study. However, the aim of this study is not to explore the accuracy and properties of different optimization approaches. Rather, we present a forecasting framework with different components and methods, which can easily be substituted with others. To enable readability of this paper, we present a summary of the method in this section and additional details in the SI.

Nelder-Mead simplex is a direct search method that attempts to minimize functions of real variables using only function evaluations without any derivatives. The minimized objective function representing differences in the daily infected counts is given by:
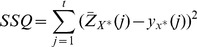
(1)

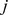
 indicates a single day and 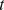 is the day on which the epidemic curve is predicted. In this study, 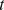 equals days 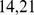, and 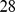. 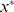 is the true parameter set and 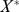 is a solution found by SIMOP. 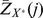 is a realization (simulation) of the curve generated by the parameter set 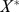 and 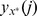 represents the estimated infected count on day 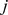 with parameters 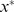.

Each parameter set contains a disease transmissibility value, an incubation period and infectious period distribution. The range of possible days for the incubation and infectious period distributions are fixed as shown in Table 1. These ranges are based on parameters used in published studies for seasonal influenza [26] and the serial interval of the 2009 pandemic [11, 57].

The algorithm proposes 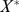 in a similar format as 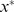 containing one value for transmissibility, in addition to four probability values for the incubation distribution and five probability values for the infectious distribution (Table 1). The probabilities must be non-negative and sum to one independently for the incubation and infectious periods. We therefore modify the Nelder-Mead algorithm by introducing conditions, which reinforce this requirement. See the SI for more information on the modified algorithm.

Each parameter set and its relative SSQ value corresponds to a vertex in a simplex. During the optimization process, the Nelder-Mead algorithm proceeds through recursive updates of the simplex vertices via a series of four basic operations: reflection, expansion, contraction and shrinkage. At each step of the Nelder-Mead algorithm, one of the formerly mentioned operations is used to generate a new parameter set that replaces a vertex in the simplex representing the parameter set with the worst SSQ value. After each update, epidemics are simulated using the new parameters and the objective function is evaluated. The next appropriate operation is selected based on the ranking (smallest to largest) of the new SSQ value relative to the values at the other vertices.

For a function of 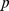 variables (parameter values), Nelder-Mead maintains 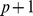 vertices forming a polytope. As earlier mentioned, there is a single transmissibility value, four possible incubation period durations and five possible infectious period durations (Table 1),which implies 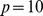. We therefore need eleven initial parameter sets. The dimension of the polytope always remains the same; containing 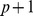 vertices. The algorithm converges if *RelDiff* is less than or equal to the relative tolerance. *RelDiff* which represents the relative difference between the vertex with the maximum SSQ and that with the minimum SSQ is defined as:
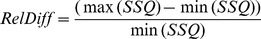
(2)


After carefully studying the convergence of the algorithm and trying several relative tolerance values, we fix the relative tolerance at 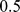. The parameter set with the smallest SSQ values at convergence is used in forecasting the epidemic curve. See references [38], [39] for additional details on the Nelder-Mead simplex method.

#### Steps iii: Simulating epidemics

As stated an individual-based model is used in simulating epidemics. Individual-based network models in epidemiology have recently garnered much attention for their advantage of being able to closely mimic realistic social networks over traditional differential equation-based disease models that assume homogeneous mixing [6], [40]. The individual-based model used in the simulations was formerly described in [31]. This and similar models have been used in several published studies [3], [6], [29], [41]. Since the creation of the individual-based model is not a novel aspect of this work, we present a brief description. Additional details are presented in the SI file.

In brief, the model is divided into two parts: a time varying social contact network and a disease model describing disease transmission between individuals and disease progression within individuals. The synthetic social contact networks are generated from a hierarchical composition of data-driven stochastic processes. First, baseline populations are synthesized based on socio-demographic statistics from the United States Census. Next, mobility patterns from a nationwide household survey and land use data are used to estimate contact networks for different regions.

In addition to demographic information, each individual is assigned an activity schedule based on responses to a national travel survey. Activities are assigned based on age, household structure and geographical location. Individuals come in contact at different activity locations such as school, work, and daycare, resulting in disease transmission between infected and susceptible individuals. One can argue that the detailed individual-based model enables both population level analysis and analysis at other granularities.

To simulate an epidemic, a population (contact network), characteristics of a disease and initial conditions (such as duration) are specified. Each simulated outbreak is replicated several times to capture different realizations of the stochastic process of disease propagation through the network. Note, compartmental models or other aggregated models can be used in place of the individual-based model.

### Synthetic Epidemic Data

The data used in this study is simulated using the individual-based model, which attempts to capture the underlying process of disease transmission. The data is simulated under different scenarios and social networks representing different geographical populations. This initial study uses only simulated data for the purpose of exploring the method's sensitivity under different scenarios and to properly manipulate and explore different outcomes of the systems.

**Figure 2:**
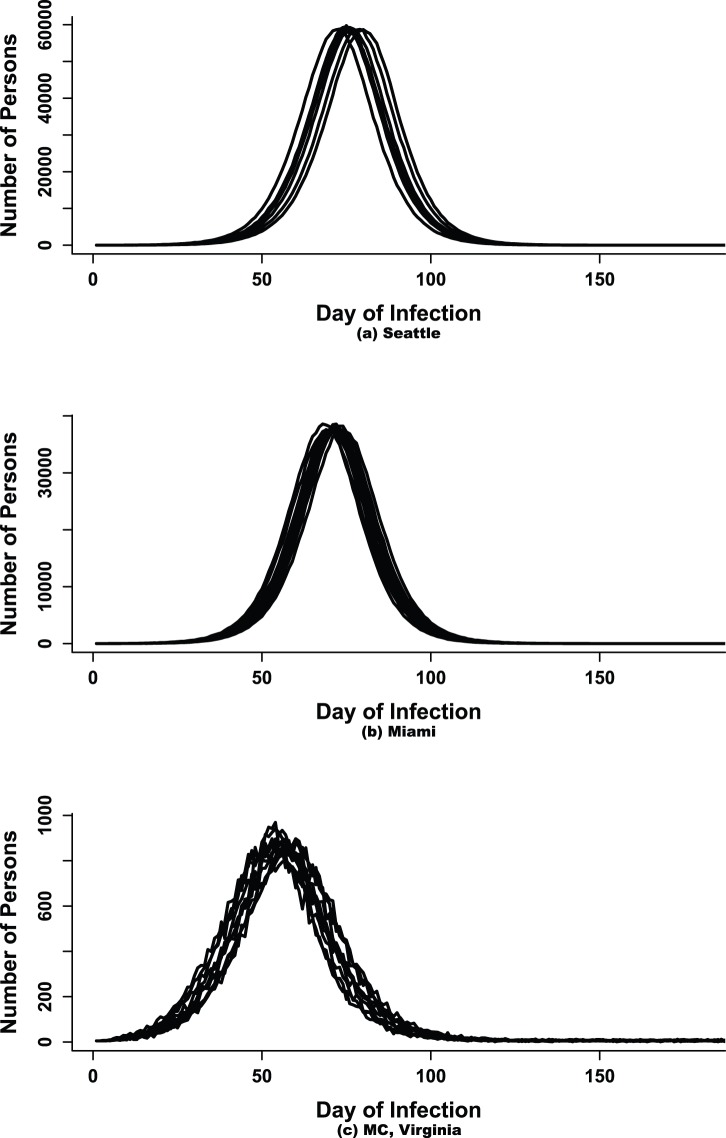
Simulated influenza incidence representing an epidemic in Seattle, Miami and MC in Virginia. The epidemics are replicated ten times by randomly selecting different individuals to initially infect. The replicated data is meant to represent the uncertainty typically observed in surveillance data.

The data is produced under two scenarios; in the first case we assume the true underlying incidence curve is unknown. We therefore produce different variations for the true curve by replicating the epidemic using different starting initially infected individuals in the population (Figure 2). Each simulation is replicated 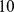 times to represent the uncertainty observed in the data collected during an epidemic due to unreported cases and differences in surveillance systems. We then forecast and measure accuracy for all three measures: peak time, peak infected and cumulative infected. In the second instance, we aim to better capture real data by distorting the daily counts of infected while maintaining the peak time. Under this scenario, we either add 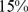 or 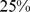 noise to the daily counts to alter the true signal. For example, let ***y*** represent the incidence data then ***y'*** 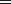 ***y*** *
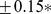
* ***y***. We focus solely on predicting the peak time.

The synthetic influenza incidence data is generated for Miami, Seattle and MC in Virginia. The synthetic populations consist of approximately 2, 3.2 and 0.16 million individuals for Miami, Seattle and MC respectively. These regions are selected due to population and demographic differences. Each epidemic representing a surveillance sample is simulated for 180-days or approximately 25-weeks for each of the synthetic networks. The epidemics are simulated using incubation and infectious period parameters which have been used in several published studies [5], [8], [29]. The simulated epidemics have a mean infectious period of 4-days, mean incubation period of 2-days and transmissibility (6.00E-5 per sec/contact time) significantly higher than that of seasonal influenza. Each epidemic is seeded by randomly selecting five individuals in the population to initially infect and on each day, in addition to infections resulting from contacts between individuals, five individuals are randomly selected and exposed. The epidemic curve is noted at the end of each simulation.

We test the forecasting approach by forecasting the epidemic curve at different time points during the epidemic. Specifically, we predict the epidemic curve on days 14, 21 and 28. We evaluate accuracy based on the predicted peak time, peak infected and cumulative infected counts. In addition, Spearman correlation coefficient and root mean squared error (RMSE) are used in assessing similarities in the temporal trend and difference between the forecasted and true epidemic curves respectively.

The accuracy of the forecast process depends not only on the Nelder-Mead algorithm but also on the objective function, and uncertainty in the available surveillance data. High levels of noise or error in the data would mask the signal of the true curve, thereby increasing the difficulty of forecast.

#### Statistical analysis

We use the optimal (smallest SSQ) parameter set at convergence to forecast the epidemic curve. The procedure is repeated 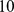 times by randomly resampling for new initial parameters from the library. In addition, for each replicate of the forecast procedure, we use a single epidemic curve from the ten replicates representing samples of the true surveillance data. Each predicted epidemic is replicated 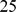 times, thereby resulting in 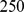 epidemic curves since the procedure is replicated 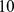 times. The means of the three public health measures (peak time, peak and total infected counts) are estimated based on the 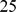 replicates of each of the predicted epidemics. This is carried out for each of the 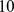 instances of the forecasting procedure. Confidence intervals are estimated around the predicted values for the public health measures. The 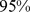 confidence intervals are calculated using the 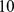 sample means. The sample means are expected to follow a t-distribution with 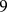 degrees of freedom. The confidence intervals are estimated as follows: 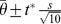, where 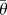 is the grand mean, 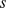 is the sample standard deviation, and 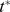 is the upper critical value for the t-distribution with 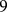 degrees of freedom.

## Results

The parameter set in Table 1 is used in simulating the epidemics across synthetic social networks for Seattle, Miami and MC in Virginia as displayed in Figure 2. The incubation and infectious period parameters have been used in several studies [3], [5], [29], [35]. Under the first scenario, each *true* epidemic is replicated 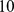 times to capture the variability that could exist in surveillance data due to misreporting and inconsistency in surveillance systems. The shape of the epidemic curve, daily counts and magnitude of the epidemics differs. This suggests that forecasts made for one region are not necessarily applicable to another. We therefore present results for each of the synthetic social networks independently. Under scenario 2, we present results for MC in VA since the overall conclusions are similar across regions.

**Figure 3:**
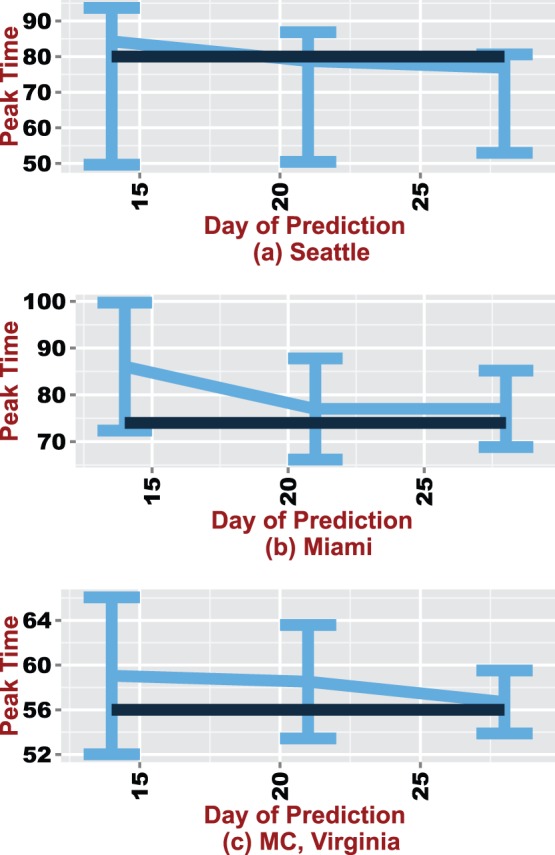
95% confidence intervals around the predicted peak time for Seattle, Miami and MC. The black line represents the true mean value.

**Figure 4:**
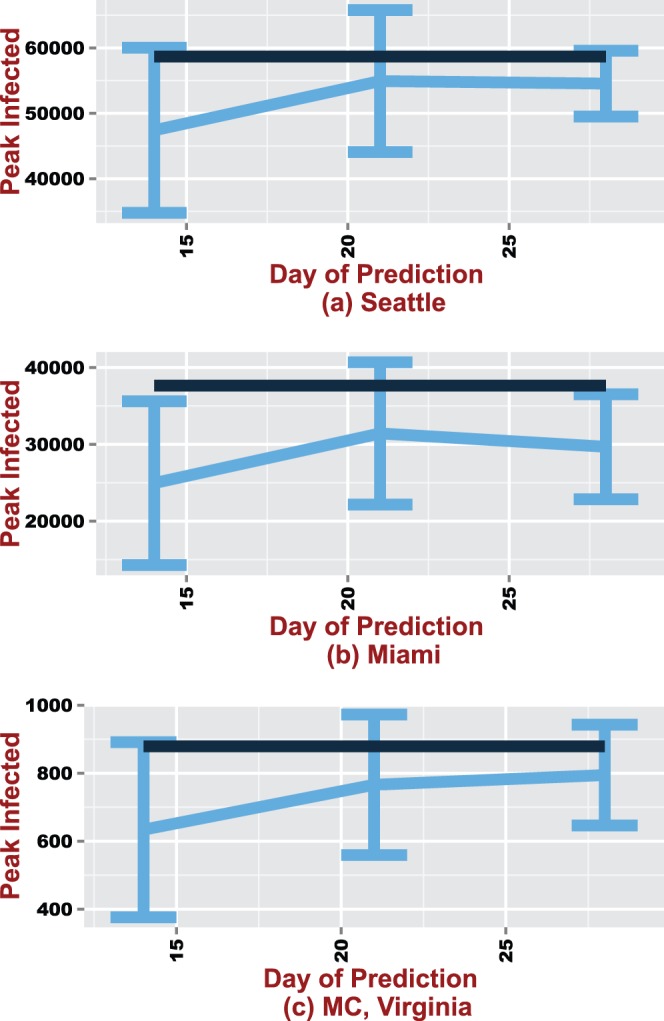
The predicted peak infected on days 14, 21 and 28 presented for Seattle, Miami and MC. The black line represents the true mean based on the ten replicates.

**Figure 5:**
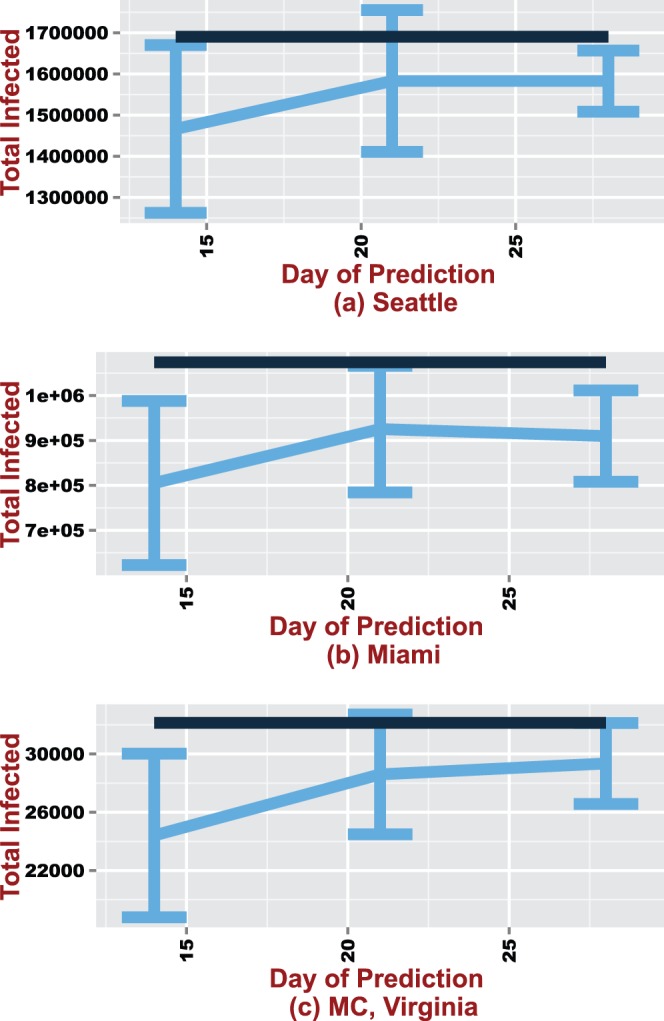
95% confidence intervals around the mean predicted total infected counts for forecasts made on days 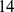, 21 and 28. The black line represents the true mean value.

The procedure is repeated 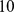 times for each forecast. Replications of the forecasting procedure enables the calculation of 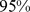 confidence intervals (CI) around the mean predicted values for the peak time, peak and total infected count. Forecasts made on day 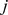 are based on available data from days 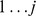. We discuss forecasts made on days 14, 21 and 28 of the epidemics. The results are presented by measures forecasted; peak time, number of individuals infected at the peak and total infected. The 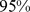 confidence intervals are also given in Figures 3, 4 and 5.

### Scenario 1

#### Peak time

As stated, forecasts made on day 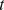 are based on data collected from days 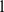 to 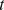. The predicted epidemics are the closest to the true epidemics during this time frame based on the norm. However, after day 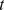, the predicted epidemics are likely to deviate from the true data indicating the different trajectories the epidemic could take. As the epidemic nears its peak, the variance in the predicted epidemic curves declines. This is expected to result in smaller confidence intervals around the predicted outcomes.

The mean peak time falls within the confidence bounds on all days for all social networks (see Figure 3). As expected the width of the CIs shrink from day 14 to 28. The mean predicted peak time overestimates the estimated true mean for Miami and MC across all days. In contrast, the true estimated mean peak time is overestimated by the predicted mean only on day 14 for Seattle. Mean peak time for MC drops from day to day and appears to be moving closer to the true mean. The estimated mean peak time for MC, Miami and Seattle are respectively days 56, 74, and 80. This would imply that the approach can accurately forecast the peak within a 95% CI at least 4 weeks, 6 weeks and 7 weeks before the actual mean peak time for MC, Miami and Seattle respectively.

#### Peak infected

The peak infected is a challenging measure to forecast especially in the early stages of an epidemic since there are several possible trajectories the epidemic curve could take. However, the estimated mean peak infected counts is captured within the forecasted 95% CI on all three days for both Seattle and MC (Figure 4). The forecasts also appear to improve over time with the smallest CI length observed on day 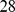. Unlike Seattle and MC, the mean peak infected fails to fall within the confidence bounds on days 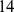 and 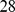. Given the mean peak day of 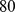 for Seattle, it is promising that the algorithm is able to capture the estimated peak infected counts within the 95% CI. Although forecasting these measures early on in the epidemic is important, the process is also extremely difficult since the epidemic is still evolving.

#### Total infected

Similar to the peak infected, the total count of infected individuals is also a difficult quantity to forecast. There are differences in the accuracy of the forecasts across the different regions (Figure 5). For Seattle, the magnitude falls within the predicted 95% CI only on day 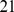. The total infected count is underestimated on all days for Miami. There is also a drop in mean predicted total infected from day 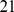 to 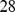. The drop in accuracy could be due to variability from different sources (Nelder-Mead algorithm, individual-based model and initial parameters) influencing the predicted outcomes. Given that day 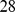 is less than halfway to the epidemics' peak, the forecasts suggest that with additional data, the true epidemic magnitude can be accurately predicted. In contrast, the total infected is correctly forecasted within the 95% CI for both days 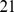 and 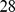 for MC. There is also an improvement in the predicted mean total infected.

#### Overall

In most cases, the forecasted mean value appears to converge to the true mean value with additional data, which reinforces the expectation that forecasts should improve as the epidemic nears its peak. In addition, the accuracy of the forecasts tend to be sensitive to the time point at which forecasting occurs as has been noted in other studies [11], [22], [24].

In general, the forecasts better capture the true trend and daily infected counts as the epidemic nears its peak for Seattle. This is supported by a drop in the root mean squared error (RMSE) from 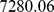 on day 14 to 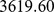 on day 28 indicating improved similarity between the true and predicted curves. In addition, the mean Spearman correlation coefficient between the true and predicted curves increased from 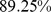 on day 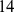 to 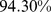 on day 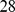.

Similar to Seattle, the forecast for Miami better captures the true trend and daily infected counts as the epidemic progresses. The RMSE dropped from 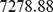 on day 14 to 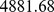 on day 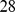 indicating improved similarity between the true and predicted curves. In addition, the mean Spearman correlation coefficient between the true and predicted curves is 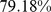 on day 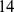 and 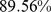 on day 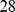.

Comparable to the observations for Seattle and Miami, the mean RMSE between the true and predicted curves is reduced from 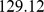 to 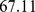 on days 14 and 28 respectively. In addition, the mean Spearman correlation coefficients between the true and predicted curves also improves from a value of 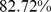 on day 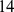 to 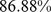 on day 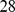. These outcomes agree with the expectation that forecasts improve as the epidemic progresses. Forecasts made for the MC synthetic population seem better compared to forecasts for Seattle and Miami. Note, all three outcomes are accurately forecasted within the 95% CI by day 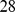.

The peak time appears to be the most suitable measure to forecast with this approach. However, in some cases, the forecasting procedure is able to correctly forecast the three public health measures with a high degree of confidence within the first six weeks of the simulated epidemics. In addition, since the accuracy of the mean predicted value consistently improves over time, this suggests that the true epidemic curve will eventually be captured during the course of the epidemic. Although there are differences in the forecasts for the different regions, a similar trend is observed in terms of accuracy. Underestimation of the total infected in the early stages of the outbreaks would suggest different approaches for controlling the spread of the epidemic for different regions. However, if such forecasts are made during the early stages of a severe epidemic, the outcomes would be useful to public health officials since even in situations where the true mean values are not captured, they are not too far off from the CIs.

### Scenario 2

**Figure 6:**
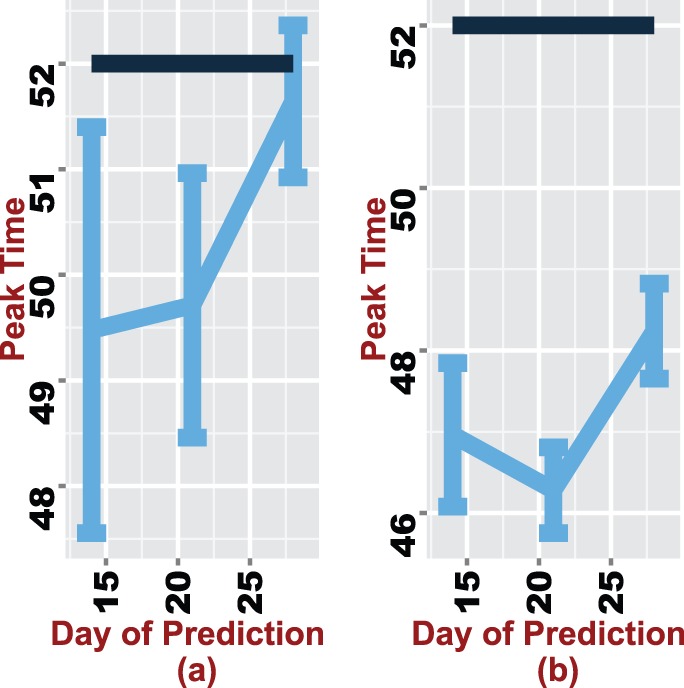
95% confidence intervals around the forecasted mean peak time on days 14, 21 and 28 for data with varying degrees of noise. A 15% and 25% error rate is added to the data used in forecasting (a) and (b) respectively.

Surveillance systems do not always capture the complete influenza incidence due to unreported cases. The collected data could therefore to be distorted. To replicate such a situation, as discussed, we add 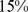 and 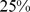 noise to the data and then proceed to forecast the peak time. Results are shown in Figure 6 for MC.

The main observation in these figures is that with additional noise in the data, predicting the peak time can be a nuisance. For Figure 6 (a), the mean predicted peak time consistently improves with additional data. The true peak is captured within the 95% CI by day 28. However, this is not the case in Figure 6 (b), the noise in the data seems to successfully mask the signal resulting in a drop in the predicted mean peak time from day 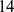 to 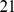. Although there is a significant improvement on day 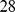, the predicted values are at least one week from the true value. In terms of accuracy one can argue that the approach performs considerable well, given that the true peak time is missed only by a week.

## Discussion

In this study, we present a method which can be used in combination with existing methods to forecast the epidemic curve during an influenza pandemic. The aims were to predict the peak time, peak infected counts and total infected counts. In addition, we also evaluated differences in forecasts across different social networks.

In some cases, the proposed method forecasted the three public heath measures within the first six weeks of the simulated epidemics. Such results would be extremely useful to public health scientists during a pandemic. Moreover, differences were observed in forecasts made across different synthetic social networks. This suggests observations made for one network are not necessary applicable to another and therefore reinforces the need for community-based forecasts [9]. By providing forecasts for a particular region, informed decisions can be made at a regional level on how to best control the disease outbreak especially when vaccinations are unavailable. Differences observed between social networks could be due to demographic differences, which have been suggested to influence epidemic spread and transmissibility [2], [42], [43]. Several studies have suggested that school children tend to impact the spread of influenza [44]–[47]. Positive correlations have also been found between the attack rate and the proportion of children within a population [42]. The percentage of the population consisting of children is approximately 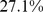 for Seattle compared to 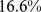 and 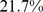 for MC in Virginia and Miami respectively. On the contrary, MC has the highest proportion of adults at 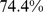. Miami has a significantly higher proportion of elderly compared to the other two social networks. Exactly how these differences in demographics influence the disease spread and consequently the forecasting process is not easily quantifiable.

### Disease Surveillance Data

Timely and accurate estimates of disease incidence are difficult to obtain during an influenza outbreak. Only a small percentage of incidence data is collected during an outbreak since most cases are unreported. Typically, ILI data are used to observe timing and other characteristics of an epidemic. Goldstein et al. [48] proposed a method for estimating incidence data from symptom surveillance data. However, due to the scarcity of the necessary data, the method was fully illustrated only on synthetic data and only partially illustrated on real outbreak data. Reliable estimates of the true incidence of influenza during an outbreak are important for this procedure. More recently, search engine query data and social media data have been suggested to augment traditional surveillance epidemic data for estimating influenza activity [49], [50]. Future research would explore the use of such alternative data sources for forecasting.

Several other issues arise when dealing with disease incidence data. Unlike the synthetic epidemic curves, ILI epidemic curves tend to be noisy. This would require adjusting the procedure to account for the uncertainty in the data which is most likely due to unreported cases. Other issues include decisions on how to initialize the epidemic model, how many new cases to introduce into the population during the epidemic and how to model data affected by non-pharmaceutical interventions. Unlike the simulated epidemics where we know the initial number of infected cases, during an epidemic this information is not readily available. One possible means of dealing with these issues involve calibrating the simulated data from the individual-based model to account for missing and unreported data. In addition, an ensemble of different forecasting techniques can be used to improve forecasts made during an outbreak.

### Optimization Procedure

Limitations in the optimization algorithm can also influence performance. In this study we used only a single optimization algorithm after comparing its performance to two other algorithms. In future studies, we would compare several algorithms to see if a single method is sufficient or whether a combination of different methods would produce better results. Also, the initial sets of parameters are crucial to the performance of the method. If initial selected parameters are similar to the true parameters, then the time to convergence would likely be shorter than if the initial parameters were farther from the true parameters. Furthermore, a study comparing the effects of different objective functions would be beneficial.

### Conclusions

The results in this study are meant to serve as an illustration that a combination of simulation and optimization methods can be used for forecasting the epidemic curve. The results are promising and indicate this approach is likely to perform well given the right model assumptions and good surveillance data. Since no existing approaches have proved infallible, this would be a reasonable method to consider for real-time forecast of the influenza epidemic curve.

## Supporting Information

Supporting Information S1 **Description of the individual-based model, the epidemic parameter search problem and the modified Nelder-Mead simplex method.**
(PDF)
