# Supplementary material for: A Simulation Optimization Approach to Epidemic Forecasting
Source: PLoS One. 2013 Jun 27;8(6):e67164. doi: 10.1371/journal.pone.0067164 (PMC3694918; doi:10.1371/journal.pone.0067164)
Supplement: Supporting Information S1 — Description of the individual-based model, the epidemic parameter search problem and the modified Nelder-Mead simplex method. (PDF) [file pone.0067164.s001.pdf]

A Simulation Optimization Approach to Epidemic Forecasting

Elaine O. Nsoesie[1], Richard J. Beckman[1], Sara Shashaani[1], Kalyani S. Nagaraj[1], Madhav V. Marathe[1,2*]

1 Network Dynamics and Simulation Science Laboratory/Virginia Bioinformatics Institute/Virginia Tech, Blacksburg, Virginia, USA

2 Department of Computer Science/ Virginia Tech, Blacksburg, Virginia, USA

∗ **E-mail: onelaine@vbi.vt.edu**

Supporting Information

Individual-based Model

Individual-based epidemiology models are used to study the effects of public health interventions and changes in individual behavior to the dynamics of infectious diseases [1]. A detailed description of the individual-based model used in simulating the outbreaks in this study is presented in [2]. The individual-based model is made up of a dynamic social contact network and a disease transmission model. The social contact network which is a synthetic representation of a particular geographical region is generated using United States Census data. The structure of the underlying network is important since interactions between agents influences how disease propagates during epidemic simulations. In this initial study, neither changes in individual behavior nor intervention strategies are explored. However, in later studies to better understand the implications of specific intervention strategies, we would forecast disease dynamics given that effective measures have been introduced to control the spread of an outbreak.

The construction of the dynamic social contact network requires two steps. In step 1 a synthetic population is created. Individuals in the synthetic population are tagged to realistic demographics while preserving the confidentiality of the original data sets. Each individual is assigned to a household using a decision tree based on demographic information (such as number of people in a household, number of children etc.) from data provided in SF3 and PUMA (Public Use Microdata Area) census files. Households are placed in realistic geographical regions. The synthetic population is statistically identical to the census aggregated at the block level. More information can be found in [3], [4] and [5].

Step 2 assigns a daily activity list with specified durations (start and end times) to each household based on several thousand responses to an activity or time-use survey. The activity templates which vary by region given factors such as the geographical location and age composition of the population, are created based on the National

Table S1: Modeling Approaches used in the Individual-based Model

**Models**	**References**
Urban Population Mobility Models	[8], [7], [9], and [10]
Natural Disease History	[11], [12], [13], [14], and [15]
Transmission Models	[13], [14], and [15]
Social Network Models	[16], [13], [17]
Types of Interventions	[18], [19], [20], and [13]

Household Transportation Survey. A gravity model and land-use data [3] are used in assigning activities to realistic locations. The addresses of non-housing locations are obtained from Dun and Bradstreet's Strategic Database Marketing Records. Presently, this modeling approach is considered the *de facto* standard in transportation science and is called activity based travel demand modeling [1].

This process results in the assignment of a minute-by-minute schedule to each of the agents in the synthetic population. The contact between agents during activities results in a realistic contact graph G_{PL}, where P is the set of people and L is the set of locations. If person $p \in P$ visits location $l \in L$, there is an edge $(p, l, label) \in E(G_{PL})$ between them with type and duration of activity recorded as *label* [1]. Edges exist between individuals in the same activity location. Synthetic individuals mimic the behaviors of real people by participating in everyday activities such as socializing, shopping etc. and multiple edges can be used between each person and the locations representing their frequency of visits. See [6] and [7] for additional information. The modeling approaches used in the individual-based as presented in [1] are given in Table S1.

After generating the social contact network, a computational model is developed to represent disease progression within individuals and transmission between individuals in the synthetic population. The disease progression is a stochastic diffusion process for which probabilistic timed transition system (PTTS) models are used. PTTS is an extension of the well known finite state machine (FSM) with probabilistic and timed state transitions (S = Susceptible, E = Exposed, I = Infectious, R = Recovered). This means that the end point of state transitions are chosen probabilistically. The interaction between individuals that are neighbors in the social contact network is represented by their PTTS. The disease can only be transmitted from an infectious node u to a susceptible node v. The probability of transmission with contact duration $w(u, v)$ is given by:

$$p(w(u,v)) = 1 - (1 - r)^{w(u,v)} \tag{1}$$

where r is the probability of disease transmission for a unit of contact time. Each infected node progresses through the succeeding transmission states where it stays for a defined period of time. The transition durations can be described using probability distributions based on the disease of interest. Several studies have been implemented to validate specific components of the model and the general approach. See [3], [16], and [13] for structural validity of these models.

To run a simulation experiment, a population (contact network), characteristics of a disease and initial conditions are specified. Each simulation is seeded with a randomly selected set of initially infected individuals and several realizations of the stochastic process of disease propagation are computed. Intervention options such as vaccination, antiviral and social distancing can be applied during the outbreak to control its propagation.

The Epidemic Parameter Search Problem

Let $G(V, E)$ denote a contact network on a population V of size $|V| = n$. Each edge $e = (u, v) \in E$ denotes contact between two nodes $u, v \in V$ and is weighted by the duration of contact $w(u, v)$. Each node $u \in V$ is assumed to be in one of four health states - susceptible, exposed, infectious, or recovered - and the set of all health states of nodes in V at the start of the simulation is given by the n-dimensional vector $\mathbf{X}_0$.

The three disease parameters used in the network-based disease model are the disease transmissibility, the incubation period distribution, and the infectious period distribution. The transmissibility of the disease (S) is defined as the probability that a node in the infectious state in the contact network infects a neighboring susceptible node for each unit of contact time. An infected node in the contact network has an incubation period of i days and an infectious period of j days with probabilities p_i^E and p_j^I, respectively, where $i = 1, \cdots, t_{\max}^E$, and $j = 1, \cdots, t_{\max}^I$. The upper bounds on the support of the incubation and infectious period distributions, $t_{\max}^E$ and $t_{\max}^I$, are obtained from literature [13, 21].

As part of the epidemic forecasting problem, we seek a set of disease parameters $\boldsymbol{\theta}^{true}$ that satisfies $\mathbf{y}(\boldsymbol{\theta})|_{\boldsymbol{\theta}=\boldsymbol{\theta}^{true}} = \boldsymbol{\alpha}$, where $\boldsymbol{\theta} = (p_1^E, p_2^E, \cdots, p_{t_{\max}^E}^E; p_1^I, p_2^I, \cdots, p_{t_{\max}^I}^I; \mathrm{S})$ denotes the set of nonnegative real valued epidemic parameter values and $\boldsymbol{\alpha} \in \mathbb{Z}^t$ denotes the epidemic curve observed up to time t for the set of disease parameters $\boldsymbol{\theta}^{true}$. The outcome of $\mathbf{y} : \mathbb{R}^d \to \mathbb{Z}^t$ ($d = t_{\max}^E + t_{\max}^I + 1$) is stochastic and can be estimated using $\mathbf{Y}_k(\boldsymbol{\theta}) = \frac{1}{k}\sum_{i=1}^{k} \mathbf{z}^i(\boldsymbol{\theta})$, where $\mathbf{z} : \mathbb{R}^d \to \mathbb{Z}^t$ is the simulated outcome of the network-based epidemic model for parameter $\boldsymbol{\theta}$, and the superscript i

indicates the ith simulated replicate.

For a fixed contact network $G(V, E)$, an initial health state $\mathbf{X}_0$ of the population V, and $\boldsymbol{\alpha}$ and $\mathbf{Y}_k$ defined as above, the epidemic parameter search problem at time t can be stated as a simulation optimization problem as follows.

$$\begin{aligned}
\text{Minimize} \quad & \|\boldsymbol{\alpha} - \mathbf{Y}_k(\boldsymbol{\theta})\| \\
\text{subject to} \quad & \sum_{i=1}^{t^E_{\max}} p^E_i = 1 \\
& \sum_{j=1}^{t^I_{\max}} p^I_j = 1 \\
& 0 \le p^E_i, p^I_j \le 1, \quad i = 1, \cdots, t^E_{\max}, \quad j = 1, \cdots, t^I_{\max} \\
& 0 \le \mathrm{S} \le 1.
\end{aligned}$$

We note that $\boldsymbol{\alpha}$ here is only one instance of the stochastic event observed for the set of disease parameters $\boldsymbol{\theta}^{true}$, but is considered as the expected epidemic outcome in the simulation optimization formulation. Also, for the purposes of this study we consider a Euclidean norm in the objective function. However, the problem formulation allows for its substitution with any other applicable norm.

Modified Nelder-Mead Simplex Method

Nelder-Mead simplex is a direct search method that attempts to minimize functions of real variables using only function evaluations without any derivatives. The Nelder-Mead algorithm proceeds through recursive updates of the simplex vertices via a series of four basic operations: reflection, expansion, contraction and shrinkage. For a function of m variables, Nelder-Mead maintains $m + 1$ vertices forming a polytope. The m variables represent m parameter sets.

At every step of the algorithm, one of the above-mentioned operations is used to generate a new parameter set that replaces a vertex in the simplex representing parameters with the worst objective value. If no better parameter set is found, all vertices are drawn halfway toward the current best vertex. This requires new simulation runs to evaluate the objective function for the updated parameter set. The dimension of the polytope always remains the same; containing $m + 1$ vertices. The algorithm converges if the relative difference between the best and

worst function values in the new polytope is less than the defined relative tolerance. The method is illustrated in Algorithm S1. Based on [22, 23] best values for the Nelder-Mead parameters are $\rho = 1$ (reflection coefficient), $x_i = 2$ (expansion coefficient), $\gamma = 0.5$ (contraction coefficient), and $\sigma = 0.5$ (shrinkage coefficient).

The modified version of the Nelder-Mead algorithm is also given in Algorithm S2. The algorithm proposes new values for the transmissibility, in addition to the probability values for the incubation and infectious period distributions. The probabilities must be non-negative and sum to one independently for the incubation and infectious periods.

Algorithm S1: Modified Nelder-Mead Simplex Algorithm with Constraints

Input: Initial parameters and surveillance epidemic curve
Output: Optimal parameter set

START:
INIT: Nelder-Mead coefficients $\rho = 1, \xi = 2, \gamma = 0.5, \sigma = 0.9$.
for $x_i \in X$: initial set of parameters in the form of vertices of size m **do**
  evaluate the objective function
end for
sort and find the best (x_l), the worst (x_h) and the second worst (x_s) set of parameters
find the centroid of the polytope ($\overline{x}$) based on the best m parameters
adjust ($\overline{x}$) using algorithm 2
repeat
  conduct REFLECTION ($x_r = (1 + \rho)\overline{x} - \rho x_h$)
  adjust x_r using algorithm 2 and evaluate $f(x_r)$
  if $f(x_l) < f(x_r) < f(x_s)$ **then**
    $x_h = x_r$
  else if $f(x_r) < f(x_l)$ **then**
    conduct EXPANSION ($x_e = (1 + \rho\xi)\overline{x} - \rho\xi$)
    adjust x_e using algorithm 2 and evaluate $f(x_e)$
    if $f(x_e) < f(x_r)$ **then**
      $x_h = x_e$
    else
      $x_h = x_r$
    end if
  else if $f(x_s) < f(x_r) < f(x_h)$ **then**
    conduct *outside* CONTRACTION ($x_c = (1 + \rho\gamma)\overline{x} - \rho\gamma x_h$)
    adjust x_c using algorithm 2 and evaluate $f(x_c)$
    if $f(x_c) < f(x_h)$ **then**
      $x_h = x_c$
    else
      replace x_h with x_r in the simplex
      for all of the parameter sets except x_l SHRINK ($x_j = x_l + \sigma(x_j - x_l)$)
      adjust x_j using algorithm 2 and evaluate $f(x_j)$
      re-evaluate $f(x_l)$
    end if
  else
    conduct *inside* CONTRACTION ($x_c = (1 - \gamma)\overline{x} + \gamma x_h$) and evaluate $f(x_c)$
    if $f(x_c) < f(x_h)$ **then**
      $x_h = x_c$
    else
      replace x_h with x_r in the simplex
      for all of the parameter sets except x_l SHRINK ($x_j = x_l + \sigma(x_j - x_l)$)
      adjust x_j using algorithm 2 and evaluate $f(x_j)$
      re-evaluate $f(x_l)$
    end if
  end if
until converged

Algorithm S2: Parameter set Adjustment

Input: A proposed parameter set
Output: Feasible parameter set
START:
set zero for every negative p_i in the set
for $i \in \{2, 3, 4, 5\}$ **do**
replace p_i with $\frac{p_i}{\sum_{k=2}^{5} p_k}$
end for
for $i \in \{6, 7, 8, 9, 10\}$ **do**
replace p_i with $\frac{p_i}{\sum_{k=6}^{10} p_k}$
end for

References

1. Barrett C, Bisset K, Leidig J, Marathe A, Marathe M (2011) Economic and social impact of influenza mitigation strategies by demographic class. Epidemics 3: 19–31.

2. Bisset K, Chen J, Feng X, Kumar VSA, Marathe M (2009) Epifast: a fast algorithm for large scale realistic epidemic simulations on distributed memory systems. In: Proceedings of the 23rd international conference on Supercomputing. ICS '09, pp. 430–439.

3. Beckman R, Baggerly K, Mckay M (1996) Creating synthetic baseline populations. Transportation Research Part A: Policy and Practice 30: 415-429.

4. Speckman P, Vaughn K, Pas E (1997a) Generating household activity-travel patterns (HATPs) for synthetic populations. Transportation Research Board 1997 Annual Meeting .

5. Speckman P, Vaughn K, Pas E (1997b) A continuous spatial interaction model: Application to home-work travel in Portland, Oregon. Transportation Research Board 1997 Annual Meeting .

6. Bowman J (2001) Activity-based disaggregate travel demand model system with activity schedules. Transportation Research Part A: Policy and Practice 35: 1–28.

7. Bowman J, Bradley M, Shiftan Y, Lawton TK, Ben-Akiva M (1998) Demonstration of an activity based model system for Portland. In: Proceedings of the 8th World Conference on Transport Research.

8. Barrett C, Beckman R, Khan M, Kumar VSA, Marathe M, et al. (2009) Generation and analysis of large synthetic social contact networks. In: Winter Simulation Conference. Winter Simulation Conference, WSC '09, pp. 1003–1014.

9. TRBC (1995-2003) 5th-9th Biennial National Academies Transportation Research Board Conferences on Application Of Transportation Planning Methods .

10. TRB (1998-2006) Transportation Research Board annual meetings .

11. Bailey N (1975) The Mathematical Theory of Infectious Diseases and its Applications. London: Griffin.

12. Elveback L, Fox J, Ackerman E, Langworthy A, Boyd M, et al. (1976) American Journal of Epidemiology 103: 152-165.

13. Halloran ME, Ferguson N, Eubank S, Longini I, Cummings D, et al. (2008) Modeling targeted layered containment of an influenza pandemic in the United States. Proceedings of the National Academy of Sciences 105: 4639–4644.

14. Hethcote HW (2000) The mathematics of infectious diseases. SIAM Review 42: 599–653.

15. Longini I, Nizam A, Xu S, Ungchusak K, Hanshaworakul W, et al. (2005) Containing pandemic influenza at the source. Science 309: 1083-1087.

16. Eubank S, Guclu H, Kumar VSA, Marathe M, Srinivasan A, et al. (2004) Modelling disease outbreaks in realistic urban social networks. Nature 429: 180–184.

17. Newman M (2003) The structure and function of complex networks. SIAM Review 45: 167-256.

18. Ferguson N, Cummings D, Cauchemez S, Fraser C, Riley S, et al. (2005) Strategies for containing an emerging influenza pandemic in Southeast Asia. Nature 43: 209-214.

19. Ferguson N, Cummings D, Fraser C, Cajka J, Cooley P, et al. (2006) Strategies for mitigating an influenza pandemic. Nature 442: 448-452.

20. Halloran E, Longini I, Cowart M, Nizam A (2002) Community interventions and the epidemic prevention potential. Vaccine 20: 3254-3262.

21. Chao DL, Matrajt L, Basta NE, Sugimoto JD, Dean B, et al. (2011) Planning for the control of pandemic influenza A (H1N1) in Los Angeles county and the United States. American Journal of Epidemiology 173: 1121–1130.

22. Bera S, Mukherjee I (2010) Performance analysis of nelder-mead and a hybrid simulated annealing for multiple response quality characteristic optimization. Proceedings of the International MultiConference of Engineers and Computer Scientists 3.

23. Nelder JA, Mead R (1965) A Simplex Method for Function Minimization. The Computer Journal 7: 308–313.
